# Ulcerative colitis combined with acute interstitial lung disease and airway disease: A case report and literature review

**DOI:** 10.3892/etm.2014.1895

**Published:** 2014-08-11

**Authors:** LISHENG XU, WEI XIAO, DEDONG MA, SHENGYU ZHOU, QINGHUI ZHANG

**Affiliations:** 1 Department of Respiratory Disease, Qilu Hospital of Shandong University, Jinan, Shandong 250012, P.R. China; 2 Department of Pathology, Qilu Hospital of Shandong University, Jinan, Shandong 250012, P.R. China

**Keywords:** lung disease, interstitial, colitis, ulcerative

## Abstract

The aim of this study was to investigate the clinical features of ulcerative colitis (UC) combined with acute interstitial lung disease (ILD). One case with acute UC combined with ILD and airway disease was reported, and the pathological diagnosis of previous cases of UC combined with ILD was retrospectively analyzed according to the corresponding literature. The present case concerned a male patient with UC who presented with dry cough and progressive dyspnea. The chest computed tomography (CT) images showed as normal on the seventh day; diffuse ground-glass shadows were observed on the 11th day and diffuse reticular, patchy, nodular shadows and lung cysts were observed on the 21st day. The results of an open lung biopsy on the 23rd day indicated pleural adhesions, and the pathologies were pulmonary fibrosis and airway inflammation. Glucocorticoid therapy was ineffective in the patient, but cyclophosphamide combined with γ globulin rapidly caused the disease to remit. A total of 24 cases with UC combined with ILD and two cases of UC combined with acute ILD were retrieved through PubMed. UC combined with acute ILD was rare in clinical practice. Patients with dry cough, progressive dyspnea and diffuse ground-glass shadows in pulmonary CT images should be closely monitored. Glucocorticoid therapy should be carefully selected and precautions should be taken against opportunistic infections of the lung. Cyclophosphamide combined with γ globulin may be an effective treatment strategy.

## Introduction

In addition to intestinal manifestations, ulcerative colitis (UC) mainly affects the joints, skin, liver and eyes. UC accompanied by pulmonary pathologies is rare in clinical settings; however, the early detection of pulmonary complications is critical for preventing the mortality of the patient (1). The rate of pulmonary complications caused by inflammatory bowel diseases is ~0.21% (2). UC can be complicated by numerous diseases, including interstitial lung disease (ILD) (3,4) airway inflammation, airway stenosis (5), pulmonary vasculitis (6), pulmonary embolism, pulmonary bullae, lung cysts (7) and pleural adhesions (8). In the literature, there were 24 cases of UC accompanied by ILD and 33 cases accompanied by airway inflammation, in which there were 2 cases of UC accompanied by airway inflammation, fibrosis of bronchi and bronchiole, and bronchitis. Whilst UC accompanied by acute ILD is rare, two cases of acute UC plus ILD were reported by Marten *et al* (3) and Chikano *et al* (4), in which high-dose corticosteroid therapy was ineffective and the patients eventually succumbed. In the present study, a case of UC accompanied by acute ILD, airway disease, lung cysts and pleural adhesions was diagnosed by the author. The disease remitted following administration of cyclophosphamide combined with γ globulin in the case previously mentioned. To further understand the clinical features of UC accompanied by acute ILD, the present case of a male with UC accompanied by acute ILD was reported and previous cases of UC accompanied by ILD that were diagnosed on a pathological basis and identified by a search of the English literature though PubMed were analyzed retrospectively.

## Case report

### Clinical data

**Figure 1:**
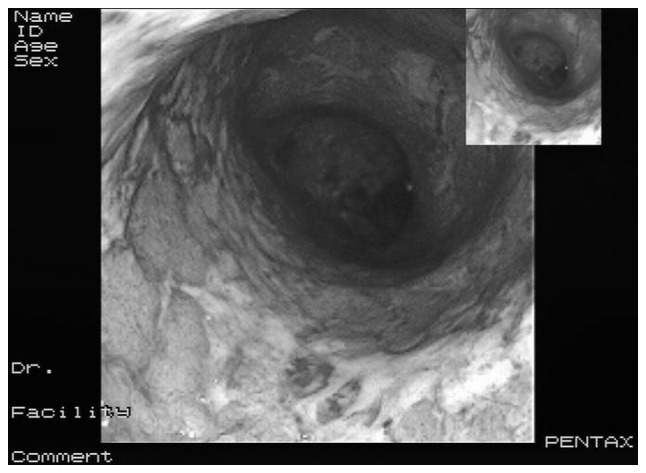
Colonoscopy showed colonic diffuse congestion, edema, a rough mucosa with fine granules and multiple shallow ulcers.

**Figure 2:**
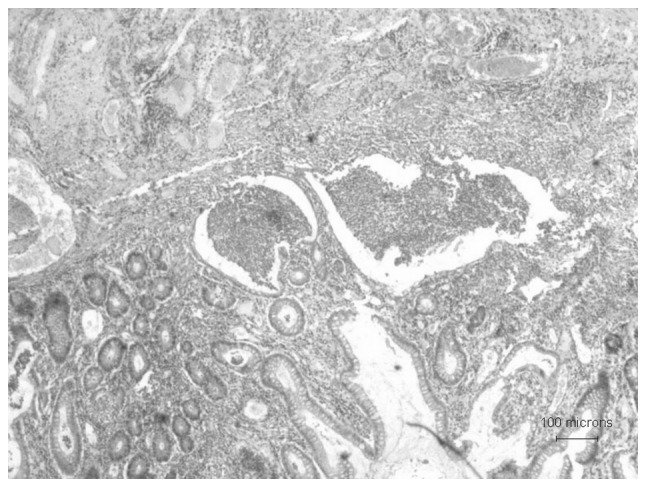
Lesions were confined to the mucosa and submucosa. Congestion, bleeding, edema and neutrophil infiltration surrounded the intestinal crypt abscesses (hematoxylin and eosin staining, magnification, ×100).

**Figure 3:**
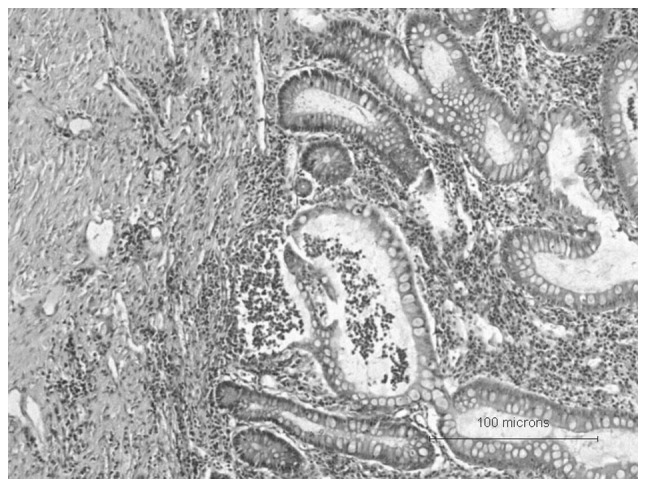
Intestinal crypt abscesses, notable neutrophil aggregation and the infiltration of chronic inflammatory cells, including lymphocytes and plasma cells, were observed, as well as mild inflammatory cell infiltration in the muscle layers (hematoxylin and eosin staining, magnification, ×400).

**Figure 4:**
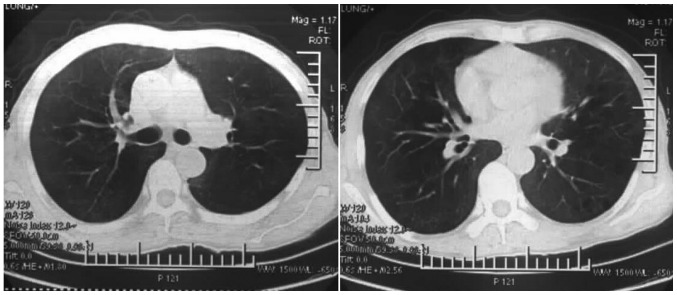
Chest computed tomography on October 15, 2007: Normal lungs.

**Figure 5:**
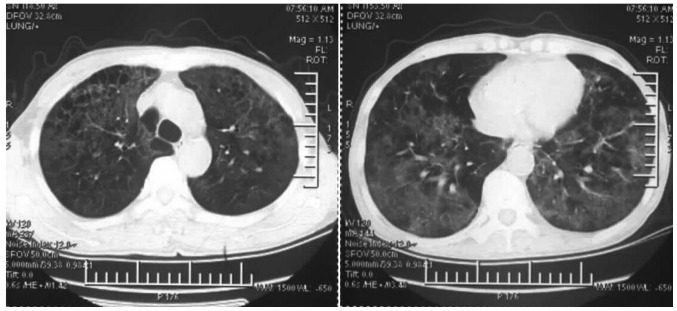
Chest computed tomography on October 19, 2007: Diffuse ground-glass shadows in the bilateral lungs and nodule shadows in the hilar region.

The patient was a male with an age of 58 years, a height of 170 cm and a weight of 65 kg. The patient had a four-year history of UC (colonoscopy images in Fig. 1 and colon biopsy histopathology images in Figs. 2 and 3), and was admitted to hospital on October 23, 2007, primarily due to dry cough and progressive dyspnea that had been present for half a month. Four years prior to the admission of the patient, the colonoscopy and pathological diagnosis had indicated UC due to chronic diarrhea and bloody stools. The patient was administered 5-aminosalicylic acid (0.5 g, four times/day) orally for three and a half years, and the disease remained in a stable condition. Four months prior to admission, the 5-aminosalicylic acid was terminated due to UC aggravation, which remitted following the administration of prednisone (30 mg/day). Half a month prior to admission, the prednisone dosage was reduced to 15 mg/day, and symptoms of dry cough and progressive dyspnea without fever appeared. The chest computed tomography (CT) was normal on the seventh day after the respiratory difficulties (Fig. 4); however, restrictive ventilatory and diffuse pulmonary dysfunction were apparent, as measured by a spirometer (Jaeger, Hoechberg, Germany). The chest CT on the 11th day showed diffuse ground-glass shadows and nodules of the hilar region in the bilateral lungs (Fig. 5). Levofloxacin, imipenem and prednisone (30 mg/day) were prescribed by the local hospital for 14 days, but were ineffective. The patient was transferred to Qilu Hospital of Shandong University (Jinan, China) on October 23, 2007 due to dyspnea. The patient had no previous history of cardiopulmonary or rheumatic diseases or other noteworthy medical history, and no history of allergies, smoking, dust inhalation or pet ownership.

### Physical examination on admission

The patient had the following characteristics on admission: Temperature, 36.8°C; heart rate, 98 beats per minute; breathing frequency, 28 times/min; and blood pressure, 107/69 mmHg. The patient was in a supine position and exhibited nervousness, shortness of breath, cyanosis of the lips and fingers, rough sounds in the lungs and feeble, moist breath. Laboratory tests were performed subsequent to admission, and a routine blood test revealed the following results: White blood cell count, 8.7×10^9^; neutrophils, 77.4% and lymphocytes, 22.6%; erythrocyte sedimentation rate, 36 mm/h; blood bacterial culture, negative; purified protein derivative test, negative; mycoplasma, chlamydia and legionella antibody, negative; cytomegalovirus and Epstein-Barr virus antibody, negative; human immunodeficiency virus antibody, negative; lactate dehydrogenase levels, 233 IU/l (a normal range is 120–230 IU/l); C-reactive protein levels, 12.4 mg/l (a normal range is 0–8 mg/l); rheumatism series antibodies and anti-neutrophil cytoplasmic antibody, negative; and complement, T-cell subsets and immunoglobulins, all within normal range. A blood gas analysis without oxygen was conducted using a blood gas analyzer (IRMA TruPoint^®^ Blood Analysis system; International Technidyne Corporation, Edison, NJ, USA), which revealed the following results: pH, 7.464; partial pressure of blood carbon dioxide (PaCO_2_), 32 mmHg; partial pressure of blood oxygen (PaO_2_), 55 mmHg; and HCO_3_^−^ levels, 25 mmol/l. In addition, the electrocardiogram and echocardiogram findings were normal. This study was conducted in accordance with the Declaration of Helsinki and with approval from the Ethics Committee of Qilu Hospital of Shandong University. Written informed consent was obtained from all participants.

### Diagnosis and treatment

**Figure 6:**
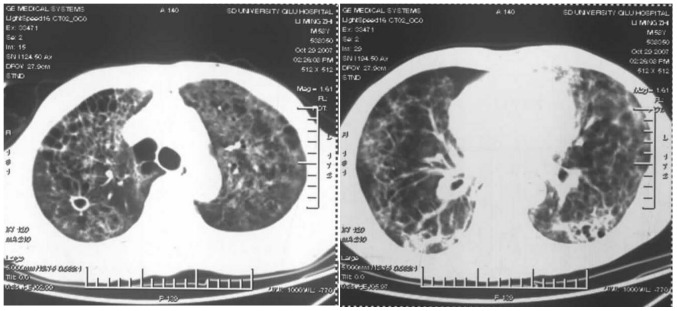
Chest computed tomography on October 29, 2007: Diffuse reticular patchy shadows; nodule shadows have become large (evident in the central region) and lobe cyst-like shadows are present in the right upper lungs.

Subsequent to admission, 3 g cefoperazone-sulbactam, 1 g vancomycin, 300 mg ganciclovir and 160 mg methylprednisolone were intravenously infused once every 12 h. However, the condition of the patient deteriorated and respiratory distress appeared. The blood gas analysis (6 l/min oxygen inhalation by mask) revealed the following results: pH, 7.35*;* PaCO_2_, 45 mmHg; PaO_2_, 57 mmHg and HCO_3_^−^ levels, 27 mmol/l. The results of the repeated chest CT scan (SOMATOM Emotion 16-slice; Siemens Healthcare, Erlangen, Germany), performed on the 21st day (seven days after admission), showed that the bilateral lungs had changed to exhibit diffuse reticular or patchy shadows, with nodular shadows on the pulmonary hilar region and cyst shadows on the right upper lobe (Fig. 6); therefore, the use of vancomycin was ceased. On the 23rd day after the disease onset, pleural fiber deposition in the visceral layer, wide pleural adhesions and nodules on the lung surface were observed in the open lung biopsy taken from the right middle lobe tissues.

**Figure 7:**
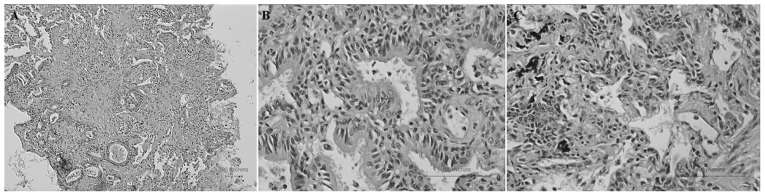
Histopathological features of the middle lobes in the right lung by open lung biopsy. (A) Lesions distributed along the small airways, bronchitis and bronchiolitis, bronchial smooth muscle proliferation and peripheral bronchial and surrounding lung tissue fibrosis can be observed (HE staining, magnification, ×100). (B) Focal bronchiolar epithelial hyperplasia and bronchiolar epithelial metaplasia (HE staining, magnification, ×400). (C) Alveolar wall thickening and alveolar type II epithelial cell proliferation (HE staining, magnification, ×400). HE, hematoxylin and eosin.

**Figure 8:**
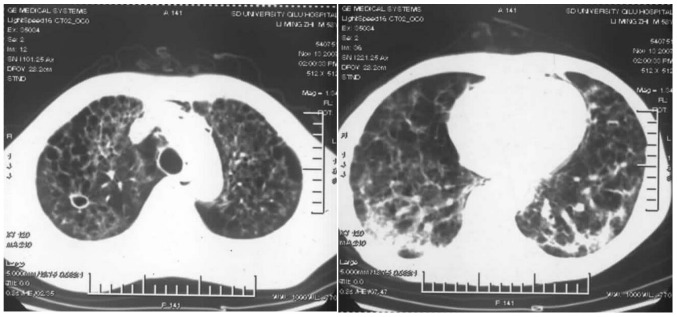
Chest computed tomography on November 10, 2007: The diffuse reticular, patchy, nodular shadows (evident in the central region) have been reduced and the cystic wall has thinned.

On the 28th day after the disease onset, the pathological reports showed fibrosis in the bronchi, bronchioles and surrounding lung tissues, and that bronchitis and bronchiolitis were also apparent. Bronchial smooth muscle hyperplasia, bronchiolar metaplasia, focal bronchiolar epithelial hyperplasia and squamous alveolar type II epithelial cell proliferation were also observed (Fig. 7). No virus inclusion bodies and trophozoite of *Pneumocystis carinii* were observed in the specimens. The disease was diagnosed to be acute UC plus ILD and airway disease by combining the clinical, imaging and pathological features. Every other day, 200 mg cyclophosphamide was intravenously administered, combined with a γ globulin intravenous injection once daily (20 g/day). On the 33rd day after the disease onset, which was the fifth day after cyclophosphamide treatment, the symptoms of dry cough and difficult breathing were significantly reduced, and the patient could carry out daily activities to a slight extent. The blood gas analysis (conducted in conditions without oxygen) revealed the following results: pH, 7.485; PaO_2_, 72 mmHg; PaCO_2_, 37.1 mmHg; and HCO_3_^−^ levels, 28 mmol/l. The repeated CT scan showed that the patchy and reticular nodules in the bilateral lungs had become smaller and the cystic wall had thinned (Fig. 8). On the 35th day after the disease onset, the condition of the patient was significantly improved and he was discharged back to the local hospital for continued administration of cyclophosphamide for 21 days (a total of 2.6 g), as the UC and lungs were in a stable condition. Prednisone (40 mg/day) was administered orally for three months subsequent to ceasing the use of cyclophosphamide. The UC relapsed when the prednisone dosage was reduced to 25 mg/day, but not the coughing, breathing difficulties or the other pulmonary symptoms. The UC then remitted following the administration of 5-aminosalicylic acid (0.5 g/day orally, four times/day). Telephone follow-up lasted for five years, during which time the UC relapsed six times and the patient was admitted and treated in local hospitals. The pulmonary condition remained stable without recurrence, but the patient eventually succumbed on November 21, 2012 due to gastrointestinal bleeding.

## Discussion

UC may be accompanied by a variety of pulmonary complications including ILD, but the complication of acute ILD is rare in clinical settings. Two patients with acute UC plus ILD reported in the literature succumbed due to ineffective glucocorticoid treatment (3,4). In the case of UC accompanied by acute ILD that was diagnosed and treated by the author, the disease remitted following administration of cyclophosphamide combined with γ globulin. To further understand the clinical diagnosis and treatment features of UC accompanied by acute ILD, cases with a pathologically based diagnosis were analyzed retrospectively according to the English literature identified by a search through PubMed.

The keywords ‘Colitis, Ulcerative/complications’ and ‘Lung Diseases, Interstitial’ and ‘Colitis, Ulcerative/complications’ and ‘Pulmonary fibrosis’ were used to search PubMed. Studies not published in English and those in which the disease was caused by drug-induced factors were excluded. According to the pathological diagnosis, 24 cases with UC complicated by ILD (six females and 10 males; age range, 13–70 years; mean age, 43±15.35 years) were selected. In eight cases the gender and age were not reported, including two cases with acute ILD insensitive to glucocorticoid therapy. Of these two cases, one patient with idiopathic interstitial pneumonia (4) succumbed 75 days after the disease onset due to opportunistic infections in the lungs, and one patient with the usual type of acute interstitial pneumonia and acute exacerbation (3) succumbed three months after the disease onset. The keywords ‘Colitis, Ulcerative/complications’ and ‘Bronchitis’, ‘Colitis, Ulcerative/complications’ and ‘Bronchiolitis’ and ‘Colitis, Ulcerative/complications’ and ‘Lung Diseases, Obstructive’ were then used to search PubMed. Studies not published in English and those in which the disease was caused by drug-induced factors were excluded. According to the pathological diagnosis, 33 cases of UC with concurrent airway disease were selected, including two cases (9,10) in which pathology showed airway inflammation, bronchial fibrosis and bronchial stenosis.

Twenty-four cases of pathologically diagnosed UC complicated by ILD retrieved in this study were assessed for certain characteristics, including the association between UC activity and the onset of ILD and the time and duration of the ILD*.* Among the 24 cases, 10 (11–13) did not report the order of the ILD and UC incidences. In 12 cases (12/14, 85.71%) the ILD occurred 1–15 years after the diagnosis of UC (3,4,7–9,14–18); this included only five cases in which the ILD occurred during active UC (5/12, 41.67%) (3,4,14,15). In two cases it was reported that the ILD occurred prior to the UC (17,19); in one of these cases (19) the ILD occurred immediately subsequent to the UC relapse, indicating that UC activity may be associated with the incidence of ILD.

Inducing factors were also examined, including whether the UC colectomy induced the ILD. Only four out of the 24 cases mentioned colectomy, including two cases (2/4, 50%) in which the ILD occurred subsequent to the colon resection (3,18), suggesting that the colectomy may be a stimulus for the occurrence of UC complicated by ILD. The two cases in which the ILD occurred prior to the colectomy were in different situations. McKee *et al* (7) reported one case in which the ILD occurred in the UC stable phase 10 years after the onset of the UC. Colectomy was then performed due to the recurrence of the UC; however, the ILD then continued to progress until mortality, suggesting that the colectomy may have aggravated the existing ILD condition. However, Isenberg *et al* (8) reported a case with a contrasting condition; this patient had been diagnosed with UC for 15 years and exhibited lung infiltrate in the bilateral lungs during the UC stable period, but had no pulmonary symptoms. The open lung biopsy diagnosis revealed diffuse vasculitis and interstitial pneumonia. Without the use of corticosteroids, the chest radiograph on the 10th day after colectomy showed pulmonary infiltrate absorption, and the chest radiograph then returned to normal.

Another inducing factor assessed was smoking history. This was not reported in 17 cases (17/24, 70.8%), and only two of the remaining seven cases (2/7, 28.6%) (3,14) had a smoking history. Smoking history has therefore not been determined to be a predisposing factor of ILD.

The clinical manifestations of the cases were next assessed. With regard to the clinical symptoms, seven patients exhibited dry cough (7/24, 29.2%) (7,9,13,16–19); two, sputum (2/24, 8.3%) (15,18); nine, shortness of breath and difficulty breathing (9/24, 37.5%) (3,7,9,13,14,17–19); four, pleuritic chest pain (4/24, 16.7%) (7,9,14,16); four, fever (4/24, 16.7%) (4,15,16,18); one, night sweats (1/24, 4.2%) (16); two, fatigue (2/24, 8.3%) (3,13); two, weight loss (2/24, 8.3%) (13,15); one, arthralgia (1/24, 4.2%) (16); and 10, no respiratory symptoms (10/24, 41.7%) (8,12,14). Symptoms of dry cough, shortness of breath and difficulty breathing were the most common in clinical settings. When UC is accompanied by acute ILD, precautions should therefore be taken to avoid the aggravation of progressive acute dyspnea (3). Patients with UC but without respiratory symptoms should also not be disregarded (12). With regard to the clinical signs assessed in the cases, one patient exhibited low lung breath sounds (1/24, 4.2%) (18); three, moist rales (3/24, 12.5%) (3,7,19); two, crepitus (2/24, 8.3%) (13,17); one, expiratory wheezing (1/24, 4.2%) (13); and one, clubbing (1/24, 4.2%) (19). With regard to lung function, restrictive pulmonary dysfunction was mentioned in four out of the 24 cases (3,7,9,13). Where open lung biopsy or thoracoscopy was visible by eye, two cases (8,16) of UC concurrent with ILD had pleural fibrosis and pleural adhesions and one case (7) had diffuse cystic lung lesions, suggesting that the lung lesions were diverse in cases of UC accompanied by ILD. The assessment of pathogenesis revealed that 22 cases (22/24, 91.7%) had chronic disease. Only two cases had acute disease (2/24, 8.3%) (3,4); however, the disease progressed rapidly and severely in these two cases and the patients eventually succumbed.

The evaluation of drug treatment revealed that glucocorticoids were used in 13 cases (3,4,7,9,13,14,16–19) and not in one case (8). Drug treatment was not reported in 10 cases. Out of the 11 chronic cases using glucocorticoids, the drugs were revealed to be effective in 10 cases (10/11, 90.9%) and ineffective in only one case (1/11, 9.1%) (7). By contrast, high-dose corticosteroid therapy was ineffective in the two cases with acute onset (including one case with concurrent opportunistic infections) (3,4), suggesting that glucocorticoids remain the preferred effective drug for cases of chronic disease, but that they should be selected with caution and vigilance for pulmonary opportunistic infections in cases of acute onset disease. Cyclophosphamide and γ globulin are typically used in the treatment for rheumatic diseases in clinical practice, but were not administered in the retrieved 24 cases of UC concurrent with ILD.

With regard to the prognosis of the cases, only one out of the 22 cases with chronic onset disease (7) (1/22, 4.5%) succumbed, while both of the two cases with acute onset disease succumbed (3,4). UC accompanied by acute ILD therefore requires early diagnosis for effective measures to be taken. The assessment of follow-up times indicated that, in addition to three cases of mortality during hospitalization, 10 cases were not followed-up. Out of the 11 cases where follow-up was conducted, the duration ranged between four weeks (9) and six years (16), with an average of 21±21.9 months.

Imaging findings from chest X-ray or CT scans revealed that one patient exhibited diffuse ground-glass shadows (1/24, 4.2%) (3); three, multiple pulmonary infiltrate shadows (3/24, 12.5%) (8,9,17); three, diffuse interstitial fibrosis (3/24, 12.5%) (7,13,18); three grid-like shadows (3/24, 12.5%) (3,13,19); one, alveolar shadows (1/24, 4.2%) (3); three, traction bronchiectasis (3/24, 12.5%) (3,7,14); three, multiple nodules (3/24, 12.5%) (14–16); one, pulmonary consolidation (1/24, 4.2%) (9); one, air bronchogram (1/24, 4.2) (15); two, cavity disease (2/24, 8.3) (14,15); and one, pleural effusion (1/24, 4.2%) (9). Diffuse interstitial fibrosis, multiple nodules, reticular patterns, honeycombing, traction bronchiectasis and a number of other symptoms were common and showed that the ILD lesions were not in the early stage. The diffuse ground-glass shadows indicated that the pulmonary lesions were in the acute exudative phase (3); therefore, precautions against acute ILD must be taken if these shadows are identified.

Following the assessment of clinical manifestations, the histological types and the methodology used to obtain the pathological specimens were examined. Firstly, the pathological types of the cases were determined. Among the 24 cases, eight patients exhibited alveolar septal fibrosis (8/24, 33.3%) (12); three, fibrosing alveolitis (16,17,19); two, diffuse interstitial pulmonary fibrosis (2/24, 8.3%) (17,18); one, idiopathic interstitial pneumonia (1/24, 4.2%) (4); one, usual interstitial pneumonia with acute exacerbation (1/24, 4.2%) (3); two, cryptogenic organizing pneumonia (2/24, 8.3%) (14,16); three, Wegener’s granulomatosis (3/24, 12.5%) (16,20); two, sarcoidosis (2/24, 8.3%) (11,15); one, interstitial pneumonia and diffuse vasculitis (1/24, 4.2%) (8); one, bronchitis and bronchiolitis with peribronchiolar fibrosis and stenosis (1/24, 4.2%) (9); one, interstitial pneumonia with necrotizing bronchiolitis and bronchiectasis (1/24, 4.2%) (13); and one, pulmonary interstitial fibrosis with bronchiectasis and organizing lipid pneumonia (1/24, 4.2%) (7). In the majority of the 24 cases, only one pathological type was apparent (20 cases, 20/24, 83.3%) and there were four cases in which two or more pathological types were apparent (four cases, 4/24, 16.7%) (7–9,13) were rare. With regard to the methods used to obtain the pathological specimens, the majority of the specimens were obtained using transbronchial lung biopsy (12 cases, 12/24, 50%) (4,12,17,18) and open lung biopsy (7 cases, 7/24, 29.2%) (7–9,11,13,16,19). In three cases the specimens were obtained by thoracoscopic biopsy (3/24, 12.5%) (14,16), one by autopsy (1/24, 4.2%) (3), and one by mediastinum lymph node biopsy (1/24, 4.2%) (15). Of these methods of obtaining specimens in the 24 cases, all transbronchial lung biopsies were performed prior to 2001 and the open lung biopsies were conducted prior to 2003. Two of the three thoracoscopy procedures (2/3, 66.6%) were performed in 2010. The clinical data of the 24 cases showed that biopsy specimens obtained from the transbronchial lung biopsy were too small to assess the fibrosis and degree of inflammation in the lung tissues, but for light injuries this remains a common screening method used in clinical practice. Taking specimens from open lung biopsies was ideal, and showed important value for clarifying the ILD type, but with heavy injury the diagnosis method was often used only when other inspection methods were intolerable to patients with severe ILD (7). The trauma induced by thoracoscopic surgery was small, and the procedure had similar diagnostic value to the open lung biopsy; thoracoscopic surgery may therefore become an important tool for the future diagnosis of ILD.

In the retrieved cases of UC concurrent with airway inflammation, the pathology of two cases (9,10) showed peribronchial fibrosis and bronchial stenosis, with pathological features similar to those of airway-centered interstitial fibrosis. Churg *et al* (21) reported that cases of central airway fibrosis exhibited a number of common characteristics. Firstly, the cause of the fibrosis was unclear, although several cases had a history of inhalation exposure. Secondly, the patients presented with a chronic cough and progressive dyspnea with restrictive ventilatory dysfunction, and the chest CT showed bronchial and vascular fibrosis, and interstitial infiltrates that were primarily located in the central hilar region. Thirdly, chest computed tomography demonstrated peribronchovascular fibrosis and interstitial thickening. In addition, subpleural focal pulmonary fibrosis was apparent, bronchioles were often narrow and twisted, without occlusion, and the interstitial airway walls and the mesenchyme were occasionally infiltrated by chronic inflammatory cells. Finally, corticosteroids and bronchodilators had poor effects.

Of the remaining retrieved cases, one patient with UC described in 1987 by Wilcox *et al* (10) had progressive exertional dyspnea; the chest X-ray showed a diffuse prominence of pulmonary vessels, and lumen stenosis and irregularities caused by submucosal concentric fibrosis of the respiratory bronchioles appeared in the open lung biopsy findings. One patient with a 13-year history of UC presented with dry cough, shortness of breath, pleuritic chest pain and restrictive pulmonary dysfunction (9). The chest X-ray showed right apical homogenous shadows and a small pleural effusion on the right, which developed into the peripheral consolidation in both upper lobes with air bronchograms, ill defined shadowing in both lower lobes and considerable loss of volume in both lungs after four weeks. The open lung biopsy showed acute and chronic bronchial and bronchiolar inflammatory cell infiltrate with associated peribronchiolar fibrosis. Furthermore, bronchiole stenosis appeared due to the concentric submucosal fibrous thickening. These two cases were not considered to be cryptogenic organizing pneumonia due to a lack of granulation tissue or fibrous tissue blocking the lumens of the bronchi.

In the case reported in the present study, prednisone was used due to the UC relapse, and ILD occurred following a reduction in the prednisone dosage. 5-aminosalicylic acid was ceased four months prior to the onset of the ILD, and the ILD was stabilized when 5-aminosalicylic acid was re-administered during the UC relapse. Therefore the hypothesis that ILD was caused by drug factors could be excluded according to this drug medication history.

Several different diseases can induce acute diffuse pulmonary parenchymal disease, including infectious diseases (tuberculosis, chlamydia and aspergillosis, and diseases caused by viruses, *Pneumocystis carinii* and mycoplasma), diffuse alveolar hemorrhage, left ventricular dysfunction, hypersensitivity pneumonitis, eosinophilic pneumonia, diffuse alveolar damage (histologically shown by transparent films), acute fibrinous and organizing pneumonia (fibrin and organizing pneumonia observed in the alveolus) and acute exacerbation of usual interstitial pneumonia (with pathological features of usual interstitial pneumonia and diffuse alveolar damage superimposition) (3). In one case of UC, reported by Aydoğdu *et al* (1), the patient had complicated cryptogenic organizing pneumonia due to virus infection, and the clinical manifestations were acute respiratory distress and leak syndromes. The case reported in the present study manifested as an acute course of disease, but the diseases mentioned above could be excluded based on the patient history, clinical manifestations, imaging, pathology and other laboratory tests.

The pathological features of the case reported in the present study were different from airway-centered fibrosis (with features of inhalation exposure history and bronchial lumen stenosis), cryptogenic organizing pneumonia (with features of granulation tissue or fibrous tissue causing an obstruction in the bronchiole), respiratory bronchiolitis associated with ILD (with pathological features of alveolar macrophage accumulation in the respiratory bronchiole and surrounding alveolus) with mild interstitial inflammation and fibrosis around the bronchioles (22). Therefore, these diseases reported previously could be excluded.

Numerous pathologies, including ILD, airway disease, lung cysts and pleural fibrosis and adhesions, appeared in the case reported in this study, unlike the previously reported 24 cases of UC concurrent with ILD (including the two cases with acute UC plus ILD). In terms of drug efficacy, the two cases reported in the literature with acute UC plus ILD eventually succumbed due to ineffective high-dose glucocorticoid treatment. In the case reported in this paper, an acute exacerbation also occurred following high-dose corticosteroid treatment, but the disease rapidly remitted upon the administration of cyclophosphamide combined with γ globulin, providing a successful treatment for severe cases of acute UC plus ILD and airway disease.

The combination of the clinical manifestations, imaging studies and pathological features led to the case reported in this study being diagnosed as acute UC plus ILD and airway disease. Excluding the drug-induced factors, the occurrence of UC-associated pulmonary complications may be explained by the fact that the lung, stomach and intestines all originate from gastrulation and are vulnerable to the impact of the same auto-antibodies. UC is a systemic disease, and the non-specific inflammation of the bronchial subepithelial layer and submucous layer of the colon are similar; therefore, immune disorder may be the key to its pathogenesis (23). UC and ILD are associated with circulating immune complexes, which activate lymphocytes and macrophages, release cytokines, stimulate mesenchymal cells, including the proliferation and synthesis of fibroblasts, and release collagen into the lung mesenchyme (4). The condition in the present study may have been associated with the explosive increase in circulating immune complexes following glucocorticoid reduction. The effective usage of cyclophosphamide combined with γ globulin treatment in this case may be explained by the quick reduction of circulating immune complex levels caused by cyclophosphamide and the avoidance of opportunistic infection occurrence in the lungs due to γ globulin.

The results of the retrospective analysis for the 24 cases of UC complicated by ILD and the one case reported in this study indicated that UC activity and colectomy may be associated with the incidence of ILD and that lung lesions present diversity when UC is concurrent with ILD, which is rare in clinical settings. Precautions against UC with acute ILD should be taken when there are symptoms of dry cough and progressive dyspnea and when the chest CT exhibits diffuse ground-glass shadows. Thoracoscopic surgery may become a more effective means of obtaining pathological specimens. Glucocorticoids remain the first choice drug therapy in chronic UC concurrent with ILD, but these should be selected carefully and with vigilance for lung opportunistic infections in cases of acute UC plus ILD, in which the selection of cyclophosphamide combined with γ globulin could be considered. Since the specimens in this study were small, more extensive case investigations or prospective studies are required.
